# Possible Use of the SUDOSCAN Nephropathy Risk Score in Chronic Kidney Disease Diagnosis: Application in Patients with Type 2 Diabetes

**DOI:** 10.3390/bios15090620

**Published:** 2025-09-18

**Authors:** Claudiu Cobuz, Mădălina Ungureanu-Iuga, Dana-Teodora Anton-Paduraru, Maricela Cobuz

**Affiliations:** 1 Faculty of Medicine and Biological Sciences, Stefan cel Mare University of Suceava, 13th Universitatii Street, 720229 Suceava, Romania; claudiu.cobuz@usm.ro (C.C.); cobuz.maricela@spjsv.ro (M.C.); 2 Integrated Center for Research, Development and Innovation in Advanced Materials, Nanotechnologies, and Distributed Systems for Fabrication and Control (MANSiD), Stefan cel Mare University of Suceava, 13th Universitatii Street, 720229 Suceava, Romania; 3 Department of Maternal and Child Medicine, “Grigore T. Popa” University of Medicine and Pharmacy, 16th Universitatii Street, 700115 Iaşi, Romania; dana.anton@umfiasi.ro; 4 “Sfântul Ioan cel Nou” Emergency Clinical Hospital, 720224 Suceava, Romania

**Keywords:** kidney disease, EGFR, type 2 diabetes mellitus, SUDOSCAN screening, diabetes nephropathy

## Abstract

The use of quick and non-invasive techniques for detecting chronic kidney disease (CKD) in patients with type 2 diabetes mellitus is desirable and has recently garnered attention. One of these techniques is the evaluation of nephropathy risk based on electrochemical skin conductance (ESC) measured with a SUDOSCAN device. This paper aims to evaluate the possibility of using SUDOSCANs in chronic kidney disease prediction in diabetic patients and to investigate the relationships between clinical characteristics and SUDOSCAN parameters. The number of patients with type 2 diabetes included in this study was 254. Clinical metabolic characteristics like glycated hemoglobin, total and LDL cholesterol, triglyceride, blood pressure, and creatinine were determined along with body mass index, diabetes duration, and age. The estimated glomerular filtration rate (EGFR) was calculated and patients were grouped into three CKD stages based on EGFR values. Electrochemical skin conductance in hands and feet was determined with a SUDOSCAN device. The results showed that patients with symptomatic CKD (S2 and 3) presented lower ESC values, along with lower EFGRs and higher creatinine levels. A significant positive but weak correlation (*p* < 0.05) was observed between SUDOSCAN nephropathy risk and EGFR. The general linear model indicated that the SUDOSCAN nephropathy risk score could be used in CKD diagnosis only if considering age, diabetes duration, and body mass index. The area under the curve (AUC) of the receiver operating characteristic (ROC) analysis revealed the moderate possibility of using the SUDOSCAN nephropathy risk score to predict CKD, since it was 0.61 (*p* < 0.01, 95% CI 0.54–0.68), but only if the other factors mentioned above are included. Based on the cut-off value of 59.50 identified, patients were grouped (values above and below cut-off), and the results showed that patients with a SUDOSCAN nephropathy risk score of <59.50 have lower SUDOSCAN-ESC values measured in their hands and feet, lower EGFR and higher creatinine levels. These results indicated the possibility of using SUDOSCAN as a supporting tool to identify CKD if it is correlated with other factors like age, diabetes duration, and body mass index. This is important for medical progress regarding the use of novel non-invasive technologies in identifying CKD associated with type 2 diabetes.

## 1. Introduction

According to the 11th edition of the IDF Diabetes Atlas (2025), approximately 589 million adults (ages 20–79) were living with diabetes worldwide in 2024, representing about 11.1% of the adult population. Given that over 90% of these cases are type 2 diabetes, this means around 530 million adults globally were affected by type 2 diabetes in 2024 [1]. According to the International Diabetes Federation, the number of adults (20–79 years) with diabetes in Romania in 2024 was 1.3 million [2], and this has increased over time.

In individuals with type 2 diabetes, comorbidities such as hypertension, hyperlipidemia, overweight/obesity, chronic kidney disease, and cardiovascular disease frequently co-occur, usually with a higher prevalence in older adults [3]. A study assessed the prevalence and co-prevalence of comorbidities in patients with type 2 diabetes, identifying chronic kidney disease and cardiovascular disease, particularly coronary artery disease, as the most common issues, while other conditions like heart failure, peripheral arterial disease, and cerebrovascular disease were less frequent [4]. Nephropathy in diabetes is the primary cause of end-stage renal disease. The main prognostic indicators are usually albuminuria and a reduced glomerular filtration rate [5,6]. Consequently, the early identification and the consistent monitoring of nephropathy in type 2 diabetes patients are critical research priorities in diabetes management, alongside the control of glycemia, hypertension, and dyslipidemia.

Biomarker enrichment, initially based on serum creatinine and now more commonly on estimated glomerular filtration rate (EGFR), is a key strategy for selecting patients in CKD clinical trials, allowing for gender-adjusted inclusion and targeting various stages of kidney disease [7]. Chronic kidney disease is classified into five stages based on eGFR, ranging from stage 1 (kidney damage with normal function, EGFR ≥ 90) to stage 5 (kidney failure, EGFR < 15 or on dialysis), with increasing severity and corresponding treatment needs [8]. EGFR can be used for chronic kidney disease identification, and it is systematically reported using various equations that incorporate demographic and laboratory variables such as age and serum creatinine levels [6]. However, routine serum creatinine testing in clinical practice is an invasive method that may imply significant costs. Given the need for improved, non-invasive CKD screening in type 2 diabetes patients, SUDOSCAN offers a promising solution by assessing sudomotor function through electrochemical skin conductance [9]. Thin, long, unmyelinated sympathetic C-fibers innervate sweat glands [10]. Assessing sudomotor function has been proposed as a valuable tool in diagnosing early peripheral autonomic neuropathy in diabetes [9]. Typically, this condition is evaluated through sweat function tests, which are standard but often time-consuming and require specialized training [11].

Some studies investigated the possibility of using SUDOSCAN to identify CKD or other complications of type 2 diabetes. For instance, Freedman et al. [12] demonstrated that non-invasively measured electrochemical skin conductance is strongly associated with kidney disease parameters in African Americans and European Americans with type 2 diabetes, offering race-specific thresholds with high sensitivity and good negative predictive values for identifying reduced kidney function. Another study revealed that at a cut-off SUDOSCAN diabetic nephropathy score of 59.5, the SUDOSCAN test demonstrated acceptable sensitivity and specificity for detecting chronic kidney disease in Chinese patients, indicating its utility in identifying patients at risk of impaired renal function [6]. Nica et al. [9] demonstrated that SUDOSCAN shows significant potential for the early, non-invasive detection of renal dysfunction in patients with type 2 diabetes, and its associated Nephro score can predict the risk of dialysis, making it a valuable tool for CKD screening and prognosis in Romanian patients. By confirming and expanding Nica et al.’s [9] findings in a different cohort, we aimed to strengthen the evidence that SUDOSCAN can be a reliable tool across multiple Romanian populations. This helps expand the discussion from single-center evidence to national-level applicability.

In this context, this study addresses the critical clinical need for early, non-invasive identification of chronic kidney disease (CKD) risk in diabetic patients, a population highly susceptible to morbidity and mortality from diabetic neuropathy. This paper aimed to highlight the possibility of using SUDOSCAN as an aid tool in early CKD identification in patients with type 2 diabetes in northern Romania. To our knowledge, this is the first study on the northern Romanian population. In fact, this study aimed to explore whether the established neuropathy risk score provided by the SUDOSCAN device shows any associations with CKD stages and renal function (eGFR, creatinine) in type 2 diabetic patients. For this purpose, SUDOSCAN electrochemical skin conductance (ESC) and nephropathy score were considered along with other body and biochemical characteristics. A comparison between different CKD stages was performed. In addition, the relationships between biochemical markers and SUDOSCAN parameters were investigated along with the performance of the SUDOSCAN nephropathy score to predict CKD in patients with type 2 diabetes. This study adds regional validation and supports the external generalizability of SUDOSCAN findings, providing, in addition, evidence needed to support integration into screening programs or cost-effectiveness in Romanian healthcare practice.

## 2. Materials and Methods

### 2.1. Patients

The study was carried out on 254 adult patients from the Department of Diabetes, Nutrition, and Metabolic Diseases of “Sfântul Ioan cel Nou” Clinical Hospital in Suceava, Romania. Data was collected between October 2024 and June 2025. The study was conducted in accordance with the ethical approval from the hospital’s Ethics Committee (Approval No. 20/24 April 2024). Before any analysis, written informed consent was obtained from every patient. Patients with or without specific symptomatology of nephropathy were considered. The exclusion criteria included: type of diabetes (type 1 was excluded), age (>18 years), pregnant or breastfeeding females, patients who had undergone limb amputation, had pacemakers or other implantable electronic devices, presented with open or infected wounds on the hands or feet, or had a history of epilepsy or active seizure disorders. The patients who did not provide informed consent were also excluded.

### 2.2. SUDOSCAN Measurements

The SUDOSCAN device (Impeto Medical, Paris, France) utilizes hand and foot electrodes connected to a computer for data acquisition and analysis [6], based on reverse iontophoresis and chronoamperometry [13]. The device evaluates sweat gland function. This system relies on an electrochemical reaction between sweat chlorides and stainless-steel electrodes under low direct current (<4 V) [10]. This non-invasive test requires no patient preparation. However, participants of the study were asked not to use lotions, creams, or emollients on their hands or feet before the test. Patients placed their palms and soles (high sweat gland density areas) on large electrodes for 2 min. Electrochemical Skin Conductance (ESC) (μS) was recorded. ESC is given by the ratio of the generated current to the constant applied direct current stimulus [10].

Electrochemical skin conductance (ESC) directly reflects chloride ion flow, indicating sweat gland function and its innervation by small, unmyelinated sympathetic C-fibers [14]. Based on validated thresholds, ESC values provide a clear interpretation of autonomic function: ESC > 60 μS in both hands and feet signifies normal autonomic function, while values between 40–60 μS suggest possible autonomic neuropathy. An ESC < 40 μS in either hand or foot confirms advanced autonomic neuropathy. In addition to these parameters, the SUDOSCAN device also provides a nephropathy risk score based on algorithms that include ESC values, age, height, weight, and BMI to calculate the score that estimates the current risk of kidney disease [6]. The algorithm for calculating the neuropathy risk score in this study was a proprietary algorithm provided by the SUDOSCAN manufacturer (Impeto Medical, Paris, France). The device automatically calculates this score by combining electrochemical skin conductance (ESC) values with certain patient characteristics. Our study relied entirely on the output generated by the device, without any modifications or recalculations on our part. The score integrates hand and foot ESC measurements, together with basic demographic variables (age, sex, height, and weight). It is expressed on a scale from 0 to 150, where lower values indicate a higher risk of small fiber/autonomic neuropathy. This index has been used in previous studies investigating diabetic neuropathy and related complications [6,15]. According to manufacturer recommendations, values of ≥70 indicate normal (low risk), while those of <70 suggest increased neuropathy risk.

### 2.3. Patient Clinical and Demographic Profile Analysis

Each patient received a standardized medical evaluation made by trained physicians. The following demographic and clinical data were collected: age, sex, type and duration of diabetes, body mass index (BMI), resting systolic and diastolic blood pressure (Systolic BP/Diastolic BP), and current medications. Blood pressure was measured after a 5 min rest in the supine position.

Laboratory parameters including glycated hemoglobin (HbA1c), total cholesterol, low-density lipoprotein cholesterol (LDL cholesterol), triglycerides, and creatinine were assessed through testing with certified clinical analyzers within the same hospital department indicated in Section 2.1.

Estimated glomerular filtration rate (EGFR) was calculated based on creatinine level and age, according to the following formula (Equation (1)) [16]:

(1) $$EGFR=175\times {creatinine}^{-1.154}\times {age}^{-0.203}\times 0.742\;(if\;female)$$

Three groups were formed, depending on EGFR values: the first group comprised patients with asymptomatic CKD (S1: stage one), the second comprised patients with mildly reduced kidney function (S2: stage two), and the third group was formed by patients with moderate and severe CKD (S3: stage three and more).

### 2.4. Statistical Analysis of Data

The statistical analysis of data was performed by using IBM SPSS Statistics software (trial version) and XLSTAT 2024 version. Before analysis, outliers were identified and removed using stem and leaf plots, and missing values were excluded from the computation. The comparisons between groups with different CKD stages and between groups with different SUDOSCAN nephropathy risk score (>59.50 or <59.50) were made by using ANOVA with Tukey test or Mann–Whitney test (*p* < 0.05), depending on the type of data distribution. The relationships between variables were evaluated through Spearman correlations (*p* < 0.05) and Principal Component Analysis. Principal component analysis was employed to reduce dimensionality and explore latent structures among the multiple clinical, biochemical, and SUDOSCAN-derived variables. This approach allowed for the identification of clusters of correlated measures, minimization of multicollinearity, and better visualization of whether the SUDOSCAN neuropathy risk score aligns with renal function markers across CKD stages. The effectiveness of the SUDOSCAN nephropathy score to predict CKD was assessed through a general linear model with group as the independent variable, along with age, diabetes duration, BMI, and sex as covariate independent variables, and SUDOSCAN nephropathy risk as the dependent variable. Significance level was 95%. In addition, receiver operating characteristic (ROC) analysis was performed to evaluate the accuracy of SUDOSCAN nephropathy risk to predict CKD.

## 3. Results

The characteristics of the patients included in this study are presented in Table 1. Three groups were formed based on the EGFR value: stage 1 (S1), with EGFR > 90 mL/min/1.73 m^2^ which refers to asymptomatic CKD; stage 2 (S2), with EGFR between 60 and 90 mL/min/1.73 m^2^ which indicates mildly reduced kidney function, and stage 3 (S3) with EGFR < 60 mL/min/1.73 m^2^ which suggest moderate and severe CKD [17]. The first group accounted for 44.88%, the second one 44.09%, and the last one represented 11.02% of the total patients considered. A percentage of 53.54% were men and 46.46% women aged between 20 and 82 years, with the highest charge being represented by patients between 50 and 70 years (Table 1). A total of 42.52% of the patients had a diabetes duration between 0 and 5 years, 34.25% between 6 and 15 years, and 23.23% more than 15 years.

### 3.1. Comparison Between Different CKD Stages Groups

The differences between various CKD stages in terms of SUDOSCAN parameters and clinical metabolic characteristics are presented in Table 2. Right foot ESC value registered significant differences among groups (*p* < 0.01), and the values decreased in the third CKD stage. Similar trends were observed for left foot and both hands ESC values, but the differences among groups were non-significant (*p* > 0.05). SUDOSCAN Nephropathy risk score decreased significantly (*p* < 0.05) as the CKD stage increased (from 65.61 in S1 to 55.63 in S3).

Glycosylated hemoglobin (HbA1c) decreased significantly (*p* = 0.03) in stages S2 and S3 compared to S1 (Table 2). Cholesterol total, LDL cholesterol, and triglycerides decreased as the CKD progressed. Diastolic and systolic BP were not significantly affected by CKD stage (*p* > 0.05). However, the S3 group has higher systolic BP compared to the S2 group. The creatinine level was significantly higher (*p* < 0.05) as the CKD progressed. The average EGFR for S1 was 110.90 mL/min/1.73 m^2^, for S2 it was 75.78 mL/min/1.73 m^2^, while for S3 the average EGFR was 49.30 mL/min/1.73 m^2^.

The BMI was higher in the S3 group compared to the S2 and S1 groups (Table 2). S3 group was characterized by the highest diabetes duration (10.82 years) and average age of the patients (65.89), while S2 exhibited the lowest diabetes duration (90.12 years) and S1 had the smallest age average (58.01). Only BMI and age were significantly different among groups (*p* < 0.05).

### 3.2. Relationships Between Variables

The relationships between variables were analyzed through Spearman correlations, and Principal Component analysis. Significantly strong positive correlations (r > 0.90, *p* < 0.05) were obtained between SUDOSCAN right and left foot ESC, and right and left-hand ESC values, respectively (Table 3, Figure S1, Supplementary Materials). SUDOSCAN nephropathy risk score was positively weakly correlated with all hand and foot ESC values (r > 0.30, *p* < 0.05). SUDOSCAN nephropathy risk was positively weakly correlated with EGFR (r = 0.34, *p* < 0.05) and BMI (r = 0.25, *p* < 0.05), and negatively weakly linked to the creatinine level (r = −0.33, *p* < 0.05) and age (r = −0.73, *p* < 0.05). Strong negative correlation was obtained between EGFR and creatinine level (r = −0.80, *p* < 0.05). Age was positively weakly correlated with creatinine level (r = 0.33, *p* < 0.05) and negatively with EGFR (r = −0.42, *p* < 0.05), while sex negatively affected creatinine level (r = −0.45, *p* < 0.05).

**Figure 1:**
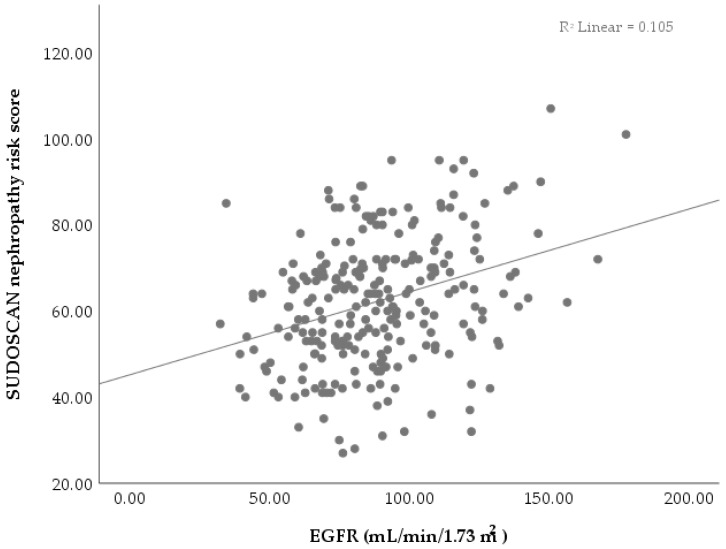
Scatter plot revealing the relationship between EGFR and SUDOSCAN nephropathy risk score.

The increase in SUDOSCAN nephropathy risk score led to better kidney function, indicated by EGFR, as suggested by the positive but weak correlation obtained (Table 3, Figure 1). While the association between EGFR and SUDOSCAN nephropathy risk score exists in our study, it is modest and does not support a strong predictive relationship. Our findings are in agreement with previous reports [6,9] and suggest the possibility of using the SUDOSCAN nephropathy risk score as a supporting tool in kidney function evaluation under limited conditions (other clinical metabolic characteristics should be considered).

Principal Component Analysis (PCA) revealed the relationships between SUDOSCAN and demographic and clinic metabolic characteristics of the patients (Table 4). The first 7 principal components were considered as the eigenvalues were > 1. These 7 principal components explained 79.02% of the total data variability.

**Figure 2:**
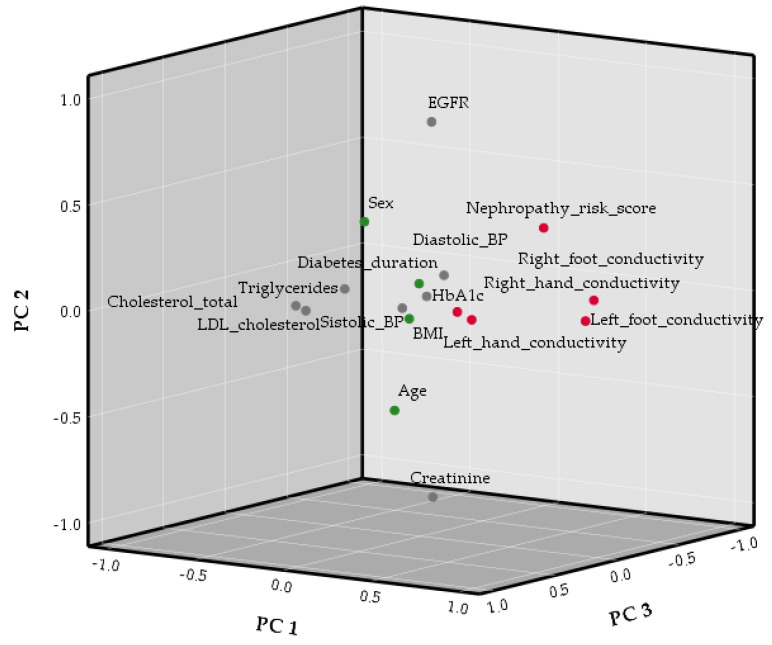
Principal component analysis plot in rotated space for the first three components.

The contribution of the variables to each principal component is presented in Table S1 in the Supplementary Materials. PC1 was associated with SUDOSCAN right and left foot conductivity and nephropathy risk score (Figure 2). PC2 was associated with creatinine and EGFR values, while PC3 was associated with total and LDL cholesterol levels. SUDOSCAN left and right hands’ conductivity were associated with PC4 (Table S1, Supplementary Materials). PC5 was associated with diabetes duration, BMI, triglycerides, and age. The systolic and diastolic BP gave PC6, while the last PC (PC7) was associated with glycosylated hemoglobin. Thus, PC1 and PC4 refer to SUDOSCAN parameters, PC2 indicates kidney function, PC3 is related to metabolism, PC5 refers to the clinical and demographical profile of the patients, PC6 indicates cardiovascular system functioning, and PC7 is related to glucose metabolism.

### 3.3. Effectiveness of SUDOSCAN to Predict Nephropathy Risk

The general linear model was used for modeling the group variable (factor), which is characterized by different EGFR limits in function of SUDOSCAN nephropathy risk score (dependent variable), with age, diabetes duration, BMI, and sex as covariate factors. The results indicated that the model is significant (*p* < 0.01). Age, diabetes duration, and BMI were the only significant variables, indicating that the SUDOSCAN nephropathy risk score could be used as an aid tool to predict CKD by considering these factors also. Age factor presented the highest effect size (partial eta-squared = 0.45) compared to the other factors included in the mathematical linear model (Table 5).

**Figure 3:**
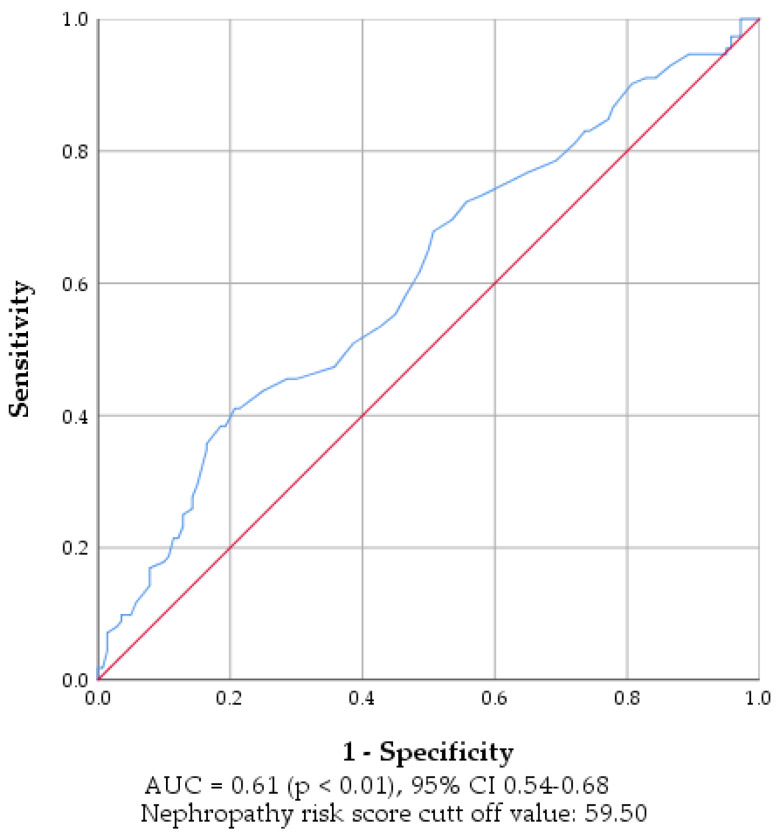
ROC curve of SUDOSCAN nephropathy risk score in identifying CKD in patients with type 2 diabetes.

The area under the curve (AUC) of the receiver operating characteristic (ROC) analysis (Figure 3) revealed a modest possibility of SUDOSCAN nephropathy risk score to predict CKD since it was 0.61 (*p* < 0.01, 95% CI 0.54–0.68), an acceptable value. The cut-off value was 59.50, considering the highest sensitivity and specificity (65.2 and 50%, respectively). Thus, the SUDOSCAN nephropathy risk score has limited standalone value for CKD prediction, as its moderate sensitivity may support early screening, but its low specificity requires additional medical investigations. Mao et al. [6] demonstrated the effectiveness of SUDOSCAN for CKD screening in Chinese patients and found that the cut-off value for the SUDOSCAN diabetic nephropathy score is 59.50. Nica et al. reported an AUC value of 0.63 (95% CI 0.563–0.696) for the SUDOSCAN Nephro-score ROC curve in patients with type 2 diabetes.

### 3.4. Comparison Between Patients with or Without Diabetic Nephropathy According to SUDOSCAN Nephropathy-Risk

The patients were grouped based on the SUDOSCAN nephropathy risk score cut-off value previously established (59.50). The comparison between the two groups is presented in Table 6. Patients with SUDOSCAN nephropathy risk score < 59.50 have lower SUDOSCAN hands and feet ESC values, EGFR, BMI, and age, while smaller creatinine levels and diabetes duration characterize patients with SUDOSCAN nephropathy risk score > 59.50. No significant differences (*p* > 0.05) were observed between groups regarding blood pressure, cholesterol levels, and glycosylated hemoglobin.

## 4. Discussion

Autonomic neuropathy affects 60% of CKD patients and, along with sustained hyperglycemia-induced protein kinase C (PKC) activation, contributes to micro- and macrovascular damage, redox stress, and diabetic microvascular complications such as nephropathy, altered blood flow, permeability changes, extracellular matrix accumulation, basement membrane thickening, and angiogenesis [17,18,19]. Small unmyelinated C fibers, vulnerable to metabolic injury and responsible for sudomotor function, can be assessed by SUDOSCAN, which measures sweat gland response to evaluate peripheral sympathetic activity [15]. This study investigated the possibility of using SUDOSCAN in CKD detection in patients with type 2 diabetes. The results showed that the prevalence of CKD was 55.11% (Table 1), which was in agreement with previous research, which reported a prevalence of CKD of 59.5% in patients with type 2 diabetes with an average age of 70.4 years [18]. Another study revealed a prevalence of CKD among a population of patients with type 2 diabetes mellitus (T2DM) standing at 53.0% [19].

The results of the present study revealed that CKD stage is related to age, diabetes duration, and BMI (Table 5). Kim et al. [18] mentioned that traditional risk factors for CKD include advanced age, obesity, high blood pressure, and diabetes mellitus. They demonstrated that a longer duration of diabetes was identified as an independent factor linked to the presence of CKD in older diabetic patients [18]. The average BMI index indicated class one obesity in patients with CKD, and increased in the S3 group (Table 2), thus supporting previous affirmations from the literature [18]. Furthermore, the increase in systolic BP in the S3 group compared to S2 confirms that hypertension can represent a risk factor in CKD. Multiple linear regression analysis performed by Mao et al. [6] revealed that the SUDOSCAN-diabetic nephropathy score, diabetes duration, age, waist-hip ratio, and hemoglobin level were significant risk factors associated with GFR. Lower HbA1c levels in S2/S3 compared to S1 may be due to more intensive treatment and closer medical monitoring in patients with advanced CKD, which can lead to apparently better glycemic control. A previous study suggested that sudomotor dysfunction shares pathogenic mechanisms with diabetic kidney disease, and Mao et al. [6] found that SUDOSCAN, using a diabetic nephropathy score cut-off of 59.5, had acceptable diagnostic value for CKD in type 2 diabetes patients, with 57.6% sensitivity, 100% specificity, and an ROC of 0.85 (95% CI, 0.76–0.93). Another paper revealed that the SUDOSCAN-Nephro score demonstrated a statistically significant ability to predict CKD in patients with type 2 diabetes in Romania, with an area under the ROC curve of 0.66, achieving 63.9% sensitivity and 33.3% specificity at a cutoff of 60.5 [9]. These results are in agreement with our findings, which revealed an acceptable AUC value for SUDOSCAN nephropathy score (0.61, *p* < 0.01) and a cut-off value of 59.50 (Figure 3). Considering the AUC value in our study, we can state that in this context, SUDOSCAN alone is insufficient as a diagnostic tool for CKD, but may serve as a supportive screening method in combination with age, diabetes duration, and BMI, particularly in resource-limited settings. The lower AUC value likely stems from interindividual variability in sudomotor function, asymmetrical neuropathy progression, or confounding factors like local skin temperature, hydration, and anatomical dominance, highlighting the crucial need for standardized measurement protocols in interpreting ESC values [9,13]. Prior studies show that elevated hydration or high ambient humidity significantly increase skin conductance, while drier or cooler conditions reduce it, thereby introducing variability [20]. Furthermore, temperature changes alter skin impedance and capacitance, which may affect ESC measurements in ways not fully controlled in our protocol [21]. These factors likely contributed to measurement noise and may partially explain the modest ROC and low specificity observed.

SUDOSCAN has shown a strong association with EGFR in patients with type 2 diabetes and an average age of 59.84 years (close to the average of 61.15 in the present study), as previously reported by Freedman et al. [22]. Furthermore, they also reported that age, gender, and BMI are significant factors affecting EGFR values [22]. This supports our significant weak correlation (Table 3, Figure 1) obtained between EGFR and SUDOSCAN nephropathy score, which is calculated by the device based on ESC, age, weight, and BMI. Similar to our results (Table 3), Chiu et al. [23] obtained a significant correlation (*p* < 0.001) between hands and feet ESC values regardless of the patient’s diabetes status. Mao et al. [24] reported that quantile regression analysis revealed a significant inverse relationship between the SUDOSCAN diabetic kidney score and EGFR, with coefficients decreasing as the diabetic kidney score percentile increased. While EGFR is the primary indicator of kidney function, it may have an inability to identify early renal dysfunction fully [25,26]. According to Mao et al. [24] the various correlations between SUDOSCAN nephropathy score and EGFR may reflect different disease severities, with more consistent results observed in patients with lower EGFR levels, indicating more severe renal dysfunction. Although most correlations observed in our study were weak, their statistical significance (*p* < 0.05) suggests that they may still reflect underlying associations; however, only the correlations between SUDOSCAN neuropathy risk and age, and between eGFR and creatinine, can be considered strong and clinically relevant (Table 3).

Considering the results of the present study and those reported in the literature, it has been demonstrated that there is a possibility to use SUDOSCAN as a supportive screening method in CKD in patients with type 2 diabetes, only if other co-factors are considered apart from EGFR (e.g., age, BMI, diabetes duration). However, in our analysis, CKD stages were grouped into 3 stages instead of 5. While this approach improved statistical power and allowed for clearer comparisons, it may have obscured important clinical distinctions between individual CKD stages. In addition, our study did not include albuminuria, a key diagnostic marker of CKD. We emphasize that while our findings support a potential association between SUDOSCAN parameters and CKD status, they should be interpreted with caution. The lack of this parameter limits the comprehensiveness of our assessment and may overstate the role of SUDOSCAN as an independent predictor. Therefore, while our results suggest that SUDOSCAN may contribute to early risk stratification, it should be viewed as a complementary tool rather than a standalone diagnostic measure.

## 5. Conclusions

Chronic kidney disease (CKD) is a pathology commonly associated with type 2 diabetes that is identified usually using biochemical biomarkers like creatinine, estimated glomerular filtration rate, and others. This study highlighted whether the established neuropathy risk score provided by SUDOSCAN shows any associations with CKD stages and renal function (eGFR, creatinine) in type 2 diabetic patients. According to the results obtained, symptomatic CKD patients have lower SUDOSCAN electrochemical skin conductance and nephropathy risk score values and higher creatinine levels. The results indicated significant weak relationships (*p* < 0.05) between SUDOSCAN nephropathy risk and EGFR. In addition, the area under the curve showed a modest prediction of SUDOSCAN nephropathy risk score for CKD in patients with type 2 diabetes and suggested that it can be used only as a supportive tool along with other screening methods. Thus, other factors such as age, diabetes duration, and body mass index should also be considered. These results underpin the use of SUDOSCAN as a practical tool for evaluating sudomotor function and supporting kidney disease diagnosis, along with other methods, in Romanian patients with type 2 diabetes.

One of the limitations of the study is related to the small sample size. Additionally, the distribution of patients across stages 3–5 was limited. Furthermore, this study employs a cross-sectional design, which does not permit the assessment of the consistency or predictive ability of SUDOSCAN over time. While standardized conditions and patient instructions aimed to minimize external influences, the inherent variability from factors like ambient temperature, skin hydration, neuropathy progression, and topical agents could still impact ESC measurements, representing a limitation that future studies should address more rigorously. SUDOSCAN can be used only in combination with biochemical analysis to obtain more precise results. Another limitation is represented by the fact that the urinary albumin-to-creatinine ratio data were not available for all patients, which restricted their inclusion in the study. This can represent an important direction for future research. Further research perspectives should also be directed to the correlations of SUDOSCAN values with other biochemical parameters from urine. Future protocols should also consider hydration, skin temperature, and neuropathy progression to refine ESC’s diagnostic performance. Furthermore, the evaluation of CKD incidence in patients with type 2 diabetes over time could provide valuable information about the possibility of using SUDOSCAN in CKD prediction and evolution. Future studies with larger samples could detail the use of SUDOSCAN in sub-stages of CKD identification.

## Figures and Tables

**Table 1:** Characteristics of the patients and groups.

Characteristic	Frequency	Percent (%)
***Group***		
S1 (EGFR > 90 mL/min/1.73 m^2^)	114	44.88
S2 (EGFR between 60 and 90 mL/min/1.73 m^2^)	112	44.09
S3 (EGFR < 60 mL/min/1.73 m^2^)	28	11.02
***Sex***		
Men	136	53.54
Women	118	46.46
***Diabetes duration***		
0–5 years	108	42.52
6–15 years	87	34.25
>15 years	59	23.23
***Age***		
<50 years	27	10.63
50–60 years	89	35.04
61–70 years	96	37.80
>70 years	42	16.54

**Table 2:** Differences between different CKD groups.

Group/ Characteristic	S1		S2		S3		*p* Value
	Mean	Std. Dev.	Mean	Std. Dev.	Mean	Std. Dev.	
***SUDOSCAN parameters***							
Right foot ESC value (μs)	78.58 ^a^	7.57	80.08 ^a^	6.69	72.07 ^b^	14.55	0.00
Left foot ESC value (μs)	79.97 ^a^	5.79	80.23 ^a^	6.21	79.00 ^a^	6.79	0.69
Right hand ESC value (μs)	64.00 ^a^	14.39	64.91 ^a^	11.97	66.11 ^a^	13.25	0.73
Left hand ESC value (μs)	64.99 ^a^	13.76	65.82 ^a^	12.60	67.85 ^a^	11.79	0.59
Nephropathy risk score	65.61 ^a^	15.80	60.37 ^ab^	14.16	55.63 ^b^	11.45	0.00
***Clinical metabolic characteristics***							
HbA1c (%)	8.47 ^a^	1.58	7.95 ^b^	1.33	7.98 ^b^	1.69	0.03
Cholesterol total (mg/dL)	181.10 ^a^	43.97	175.59 ^ab^	44.54	161.14 ^b^	38.88	0.10
Triglycerides (mg/dL)	146.87 ^a^	68.57	125.85 ^b^	53.21	146.83 ^a^	63.79	0.04
LDL cholesterol (mg/dL)	123.61 ^a^	38.62	113.37 ^ab^	35.62	100.96 ^b^	31.28	0.01
Systolic BP (mmHg)	143.76 ^a^	17.58	140.47 ^a^	15.92	143.83 ^a^	6.64	0.30
Diastolic BP (mmHg)	82.07 ^a^	10.19	80.72 ^a^	11.33	78.79 ^a^	11.38	0.32
Creatinine (mg/dL)	0.67 ^c^	0.12	0.90 ^b^	0.14	1.25 ^a^	0.22	0.00
EGFR (mL/min/1.73 m^2^)	110.90 ^a^	18.70	75.78 ^b^	8.32	49.30 ^a^	8.41	0.00
***Patient characteristics***							
BMI (kg/m^2^)	32.99 ^a^	5.75	31.44 ^a^	4.19	33.34 ^a^	5.90	0.04
Diabetes duration (years)	9.39 ^a^	9.33	9.12 ^a^	7.35	10.82 ^a^	8.34	0.63
Age (years)	58.01 ^b^	9.80	63.15 ^a^	7.40	65.89 ^a^	8.36	0.00

^a–c^: mean values followed by different letters in the same row indicate significant differences (*p* < 0.05) between groups.

**Table 3:** Spearman correlations between variables.

	Right Foot ESC Value	Left Foot ESC Value	Left Hand ESC Value	Right Hand ESC Value	Nephropathy Risk	Diabetes Duration	BMI	HbA1c	Cholesterol Total	Triglycerides	LDL Cholesterol	Systolic BP	Diastolic BP	Creatinine	EGFR	Age	Sex
Right foot ESC value value	1.00																
Left foot ESC value	0.91 **	1.00															
Left hand ESC value	0.33 **	0.40 **	1.00														
Right hand ESC value	0.30 **	0.37 **	0.90 **	1.00													
Nephropathy risk	0.62 **	0.60 **	0.37 **	0.41 **	1.00												
Diabetes duration	0.02	0.00	−0.08	−0.07	−0.21 **	1.00											
BMI	0.14	0.18 *	0.09	0.12	0.25 **	−0.14	1.00										
HbA1c	0.06	0.05	−0.02	0.01	0.16	0.25 **	0.14	1.00									
Cholesterol total	−0.03	0.00	0.01	0.06	−0.01	−0.10	−0.04	0.11	1.00								
Triglycerides	0.01	−0.04	0.03	0.09	0.14	0.00	0.14	0.11	0.39 **	1.00							
LDL cholesterol	−0.12	−0.08	−0.10	−0.08	−0.07	−0.18 *	0.01	0.08	0.75 **	0.32 **	1.00						
Systolic BP	0.02	0.08	−0.06	−0.10	−0.08	−0.03	0.16	0.01	0.06	−0.01	0.08	1.00					
Diastolic BP	0.14	0.07	−0.08	−0.12	0.05	−0.12	0.13	−0.10	0.08	0.00	0.04	0.49 **	1.00				
Creatinine	−0.12	−0.04	−0.09	−0.11	−0.33 **	0.13	−0.04	−0.05	−0.24 **	−0.03	−0.13	0.08	0.05	1.00			
EGFR	0.16	0.04	0.03	0.04	0.34 **	−0.16	−0.05	0.08	0.14	−0.03	0.15	−0.08	0.05	−0.80 **	1.00		
Age	−0.15	−0.14	−0.19 *	−0.23 **	−0.73 **	0.25 **	−0.15	−0.12	−0.12	−0.17	−0.02	0.12	−0.02	0.33 **	−0.42 **	1.00	
Sex	−0.04	−0.02	0.15	0.21 *	0.15	−0.02	0.13	0.05	0.15	0.19 *	−0.06	−0.10	−0.17 *	−0.45 **	−0.07	−0.10	1.00

*: significant at *p* < 0.05, **: significant at *p* < 0.01.

**Table 4:** Contributions of the principal components to the data variance.

Component	Eigenvalue	Variance (%)	Cumulative Variance (%)
PC1	3.72	21.88	21.88
PC2	2.60	15.28	37.17
PC3	1.92	11.31	48.47
PC4	1.67	9.82	58.29
PC5	1.31	7.73	66.02
PC6	1.13	6.62	72.64
PC7	1.09	6.39	79.02

**Table 5:** Results of the general linear model for SUDOSCAN nephropathy risk score depending on CKD stage group, age, diabetes duration, BMI, and sex.

Source	Type III Sum of Squares	df	Mean Square	F	*p*	Partial Eta Squared
Corrected Model	30,795.29 ^a^	6	5132.55	52.41	0.00	0.57
Intercept	30,145.41	1	30,145.41	307.80	0.00	0.57
Age	18,862.54	1	18,862.54	192.59	0.00	0.45
Diabetes duration	925.95	1	925.95	9.45	0.00	0.04
BMI	465.86	1	465.86	4.76	0.03	0.02
Sex	252.77	1	252.77	2.58	0.11	0.01
Group	141.10	2	70.55	0.72	0.49	0.01

^a^: R^2^ = 0.57 (Adjusted R^2^= 0.56).

**Table 6:** Characteristics of the patients with vs. without diabetic nephropathy according to SUDOSCAN nephropathy risk.

	SUDOSCAN Nephropathy Risk Score < 59.50 (G1)		SUDOSCAN Nephropathy Risk Score > 59.50 (G1)		*p*-Value
	Mean	Std. Dev.	Mean	Std. Dev.	
***SUDOSCAN parameters***					
Right foot ESC value (μs)	73.99 ^b^	9.59	80.98 ^a^	7.05	0.00
Left foot ESC value (μs)	76.50 ^b^	6.13	81.88 ^a^	5.15	0.00
Right hand ESC value (μs)	59.58 ^b^	13.36	68.37 ^a^	11.82	0.00
Left hand ESC value (μs)	61.74 ^b^	13.13	68.53 ^a^	12.23	0.00
Nephropathy risk score	48.14 ^b^	7.60	72.32 ^a^	10.01	0.00
***Clinical metabolic characteristics***					
HbA1c (%)	8.11 ^a^	1.55	8.25 ^a^	1.47	0.35
Cholesterol total (mg/dL)	172.12 ^a^	44.59	179.69 ^a^	43.30	0.21
Triglycerides (mg/dL)	129.49 ^a^	60.17	144.29 ^a^	63.68	0.05
LDL cholesterol (mg/dL)	115.09 ^a^	35.10	117.76 ^a^	38.80	0.75
Systolic BP (mmHg)	143.46 ^a^	14.65	141.51 ^a^	17.38	0.27
iastolic BP (mmHg)	79.55 ^a^	11.44	82.22 ^a^	10.31	0.09
Creatinine (mg/dL)	0.89 ^a^	0.25	0.79 ^b^	0.21	0.00
EGFR (mL/min/1.73 m^2^)	81.17 ^b^	23.86	94.23 ^a^	25.73	0.00
***Patient characteristics***					
BMI (kg/m^2^)	66.95 ^b^	8.05	56.78 ^a^	7.25	0.00
Diabetes duration (years)	12.30 ^a^	9.98	7.27 ^b^	6.15	0.00
Age (years)	30.86 ^b^	4.73	33.50 ^a^	5.25	0.00

^a,b^:different letters in the same row indicate significant differences between groups by the Mann–Whitney test (*p* < 0.05), G1/G2–groups.

## Data Availability

Data will be made available on request.

## Supplementary Materials

The following supporting information can be downloaded at: https://www.mdpi.com/article/10.3390/bios15090620/s1, Figure S1: Scatter plots showing relationships between variables; Table S1: Rotated Component Matrix.
