# Supplementary material for: Possible Use of the SUDOSCAN Nephropathy Risk Score in Chronic Kidney Disease Diagnosis: Application in Patients with Type 2 Diabetes
Source: Biosensors (Basel). 2025 Sep 18;15(9):620. doi: 10.3390/bios15090620 (PMC12467832; doi:10.3390/bios15090620)
Supplement: Supplementary file 1 [file biosensors-15-00620-s001.zip › biosensors-3791196-supplementary.pdf]

Possible Use of the SUDOSCAN Nephropathy Risk Score in Chronic Kidney Disease Diagnosis: Application in Patients with Type 2 Diabetes

Claudiu Cobuz [1], Mădălina Ungureanu-Iuga [2,*], Dana-Teodora Anton-Paduraru [3], and Maricela Cobuz [1,4]

Figure S1. Scatter plots showing relationships between variables

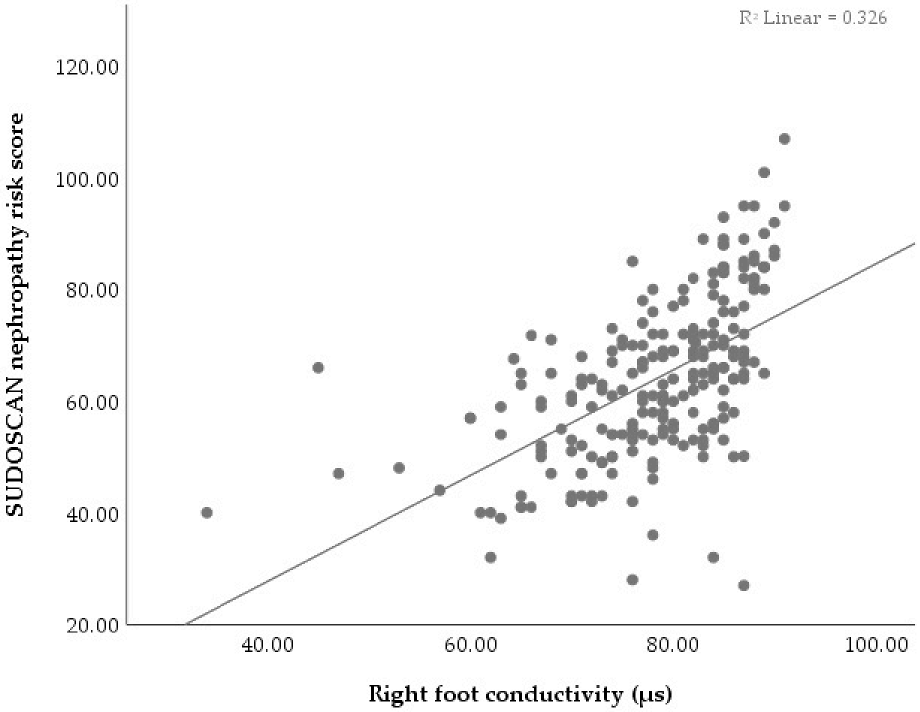

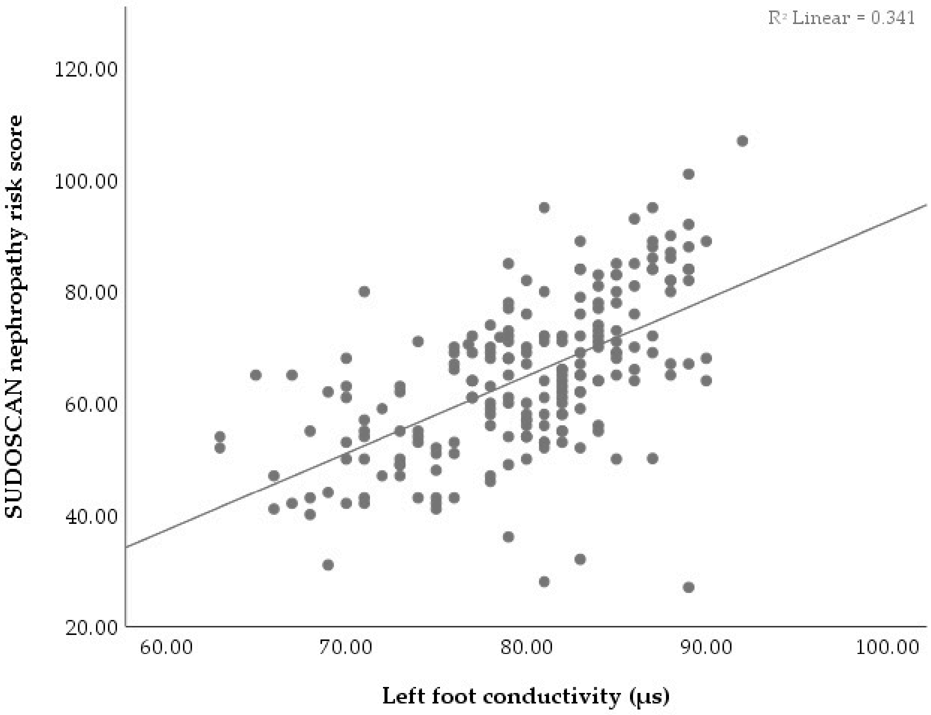
R² Linear = 0.341
120.00
100.00
80.00
60.00
40.00
20.00
SUDOSCAN nephropathy risk score
60.00
70.00
80.00
90.00
100.00
Left foot conductivity (µs)

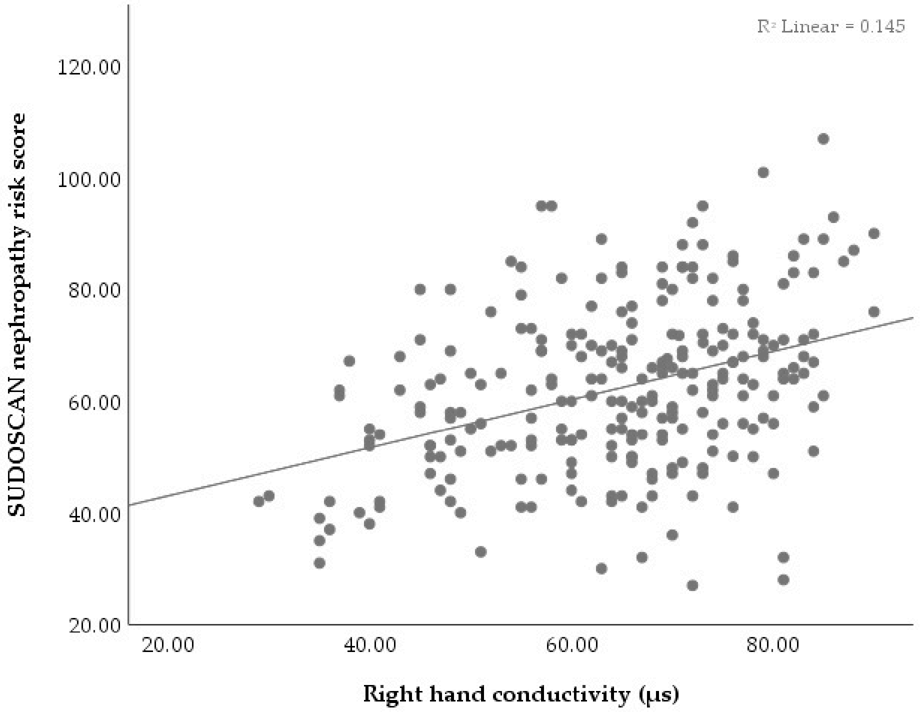
R² Linear = 0.145
120.00
100.00
80.00
60.00
40.00
20.00
SUDOSCAN nephropathy risk score
20.00
40.00
60.00
80.00
Right hand conductivity (µs)

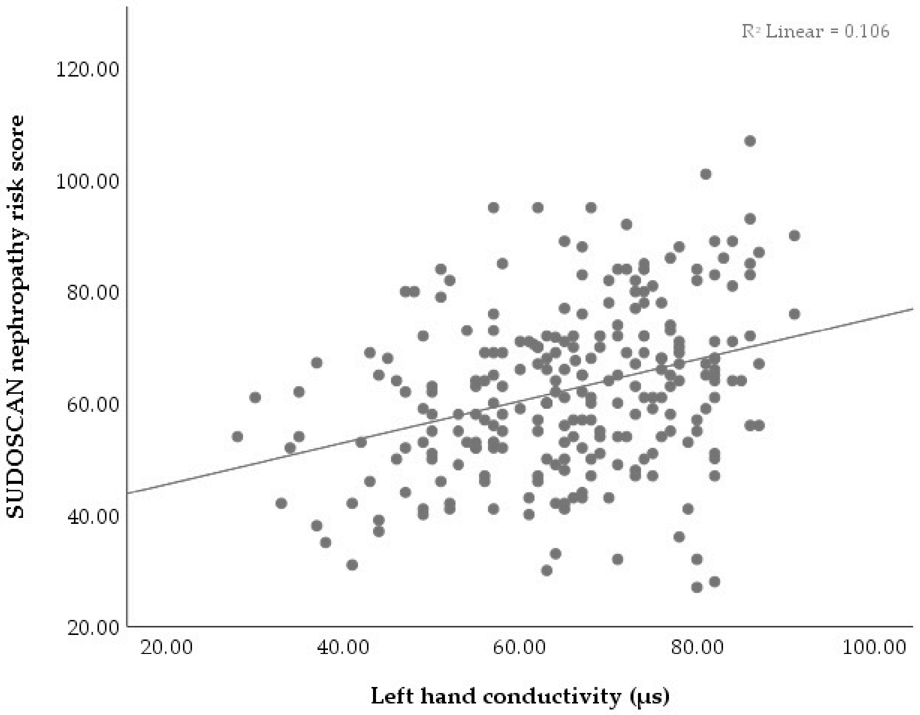

R² Linear = 0.106
120.00
100.00
80.00
60.00
40.00
20.00
SUDOSCAN nephropathy risk score
20.00
40.00
60.00
80.00
100.00
Left hand conductivity (μs)

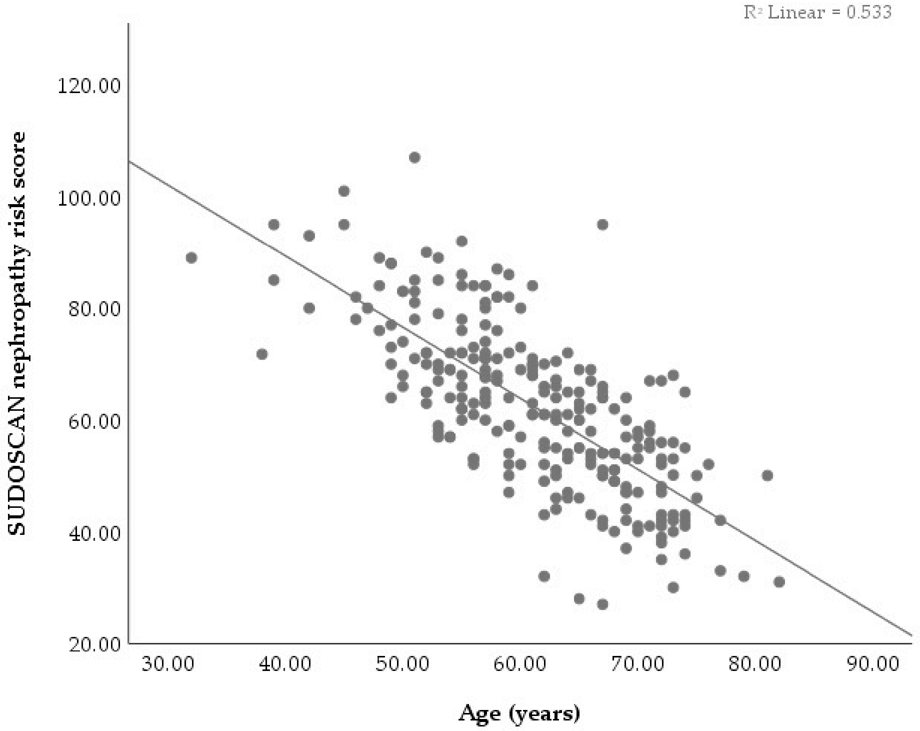

R² Linear = 0.533
120.00
100.00
80.00
60.00
40.00
20.00
SUDOSCAN nephropathy risk score
30.00
40.00
50.00
60.00
70.00
80.00
90.00
Age (years)

Table S1. Rotated Component Matrix

	Component						
	**PC1**	**PC2**	**PC3**	**PC4**	**PC5**	**PC6**	**PC7**
Right_foot_conductivity	0.93	0.08	-0.08	0.07	0.00	0.03	-0.01
Left_foot_conductivity	0.91	-0.01	-0.03	0.21	0.02	0.10	-0.02
Left_hand_conductivity	0.26	-0.07	-0.03	0.87	0.04	-0.08	-0.02
Right_hand_conductivity	0.25	-0.02	0.06	0.90	0.06	-0.11	0.00
Nephropathy_risk_score	0.69	0.41	0.00	0.13	0.45	-0.02	0.16
Diabetes_duration	-0.10	0.05	-0.12	-0.01	-0.69	-0.17	0.41
BMI	-0.08	-0.10	-0.03	0.30	0.57	0.21	0.39
HbA1c	0.05	0.03	0.03	0.01	-0.01	0.01	0.89
Cholesterol_total	-0.06	0.10	0.92	0.11	-0.06	0.16	0.01
Triglycerides	-0.01	0.14	0.60	0.10	0.40	-0.15	0.19
LDL_cholesterol	-0.04	0.08	0.88	-0.10	0.05	0.10	-0.10
Sistolic_BP	0.00	-0.01	0.16	-0.02	-0.07	0.88	0.11
Diastolic_BP	0.12	0.13	-0.01	-0.14	0.27	0.77	-0.12
Creatinine	0.00	-0.94	-0.09	-0.10	0.07	-0.05	-0.01
EGFR	0.15	0.88	0.13	-0.07	0.04	0.08	-0.04
Age	-0.24	-0.56	-0.13	0.01	-0.59	0.11	-0.15
Sex	-0.28	0.35	0.06	0.55	0.10	0.03	0.14

Extraction Method: Principal Component Analysis.
Rotation Method: Varimax with Kaiser Normalization.
a. Rotation converged in 7 iterations.
